# Protocol for measuring erythrocyte glutathione reductase activity coefficient to assess riboflavin status

**DOI:** 10.1016/j.xpro.2023.102726

**Published:** 2023-11-20

**Authors:** Damon A. Parkington, Albert Koulman, Kerry S. Jones

**Affiliations:** 1 Nutritional Biomarker Laboratory, MRC Epidemiology Unit, University of Cambridge, Cambridge CB2 0SL, UK

**Keywords:** Chemistry, Health Sciences

## Abstract

Riboflavin (vitamin B2) is a component of the co-enzyme flavin adenine dinucleotide (FAD). The activity coefficient of erythrocyte glutathione reductase (EGRAC), a FAD-dependent enzyme, is a biomarker of riboflavin status. Here, we describe a protocol for measuring unstimulated (basal) and FAD-stimulated (activated) erythrocyte glutathione reductase activity to calculate EGRAC. We describe the steps for preparing washed red blood cells and hemolysates; preparing reagents; loading, incubating, and reading the 96-well plate; and calculating the results.

For complete details on the use and execution of this protocol, please refer to Hess et al.^1^

## Highlights

• A protocol for measuring erythrocyte glutathione reductase (EGR) activity coefficient (EGRAC)
• Steps for preparing washed red blood cells and hemolysates
• Instructions for measuring unstimulated (basal) and FAD-stimulated EGR activity
• Calculations of EGRAC and quality control checks

## Abstract (editor highlights)

Publisher’s note: Undertaking any experimental protocol requires adherence to local institutional guidelines for laboratory safety and ethics.

## Graphical abstract

**Figure:**
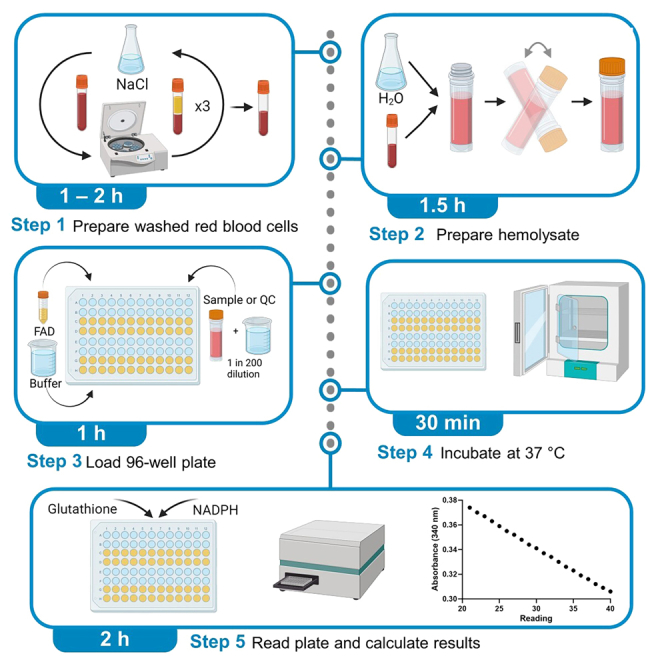

## Before you begin

### Prepare washed red blood cells

**Timing: 1–2 h**

***Note:*** The preparation of washed red blood cells (wRBC) should be completed prior to freezing of the sample.

***Note:*** Samples should be collected into a tube containing EDTA or lithium heparin (LH) anticoagulant.

1. Pre-chill centrifuge to 4°C.

***Note:*** If a refrigerated centrifuge is not available, then pre-cooling of centrifuge buckets and blocks at 4°C is recommended.

2. Mix blood tubes by inverting slowly 10 times.
3. Centrifuge the tubes at 4°C for 10 min at 3000 × *g.*
4. Remove the plasma using a pipette and discard or store for future use.
5. Remove the buffy coat from the sample using a Pastette, and discard or store for future use.

***Note:*** The buffy coat is the thin layer of white blood cells and platelets which should be just visible between the red blood cells (RBC) and the plasma after centrifugation.

6. Re-suspend the RBC in normal saline (0.9% w/v NaCl solution) to approximate the original volume of whole blood. Re-cap and gently invert the tube 10 times to mix.
7. Centrifuge the tubes at 4°C and 2000 × *g* for 10 min and, using a Pastette, remove and discard the saline supernatant.
8. Repeat steps 6 and 7 twice for a total of 3 washes or until the saline supernatant is clear.
9. Remove and discard the saline supernatant and the top 1 mm of RBC to ensure complete removal of saline.

**CRITICAL:** All the saline supernatant must be removed to ensure that only the RBCs remain in the tube.

10. wRBC should be stored at ‒70°C.

***Note:*** wRBC samples should be frozen at ‒70°C. Where this is not possible, samples should be stored at ‒20°C and transferred to ‒70°C or below as soon as possible.

### Program plate reader software

**Timing: 0.5–1 h**

11. Program the plate reader to read each well of the plate at 340 nm, 40 times at 1 min intervals, with the plate reader set to shake the plate on a moderate setting for 5 s before each read. The plate reader should be set to incubate the microplate at a constant temperature of 28°C during the read.

***Note:*** The options for shaking speed vary between different plate readers. It is recommended that a moderate setting is selected where available and that vigorous shakes, which could cause foaming, are avoided.

***Note:*** This step should be completed prior to the analysis day. It should be possible to save the program for future use.

### Prepare quality control material

**Timing: 2–4 h**

**CRITICAL:** Quality control (QC) material should be prepared to run alongside every batch to monitor batch-to-batch imprecision.

***Note:*** Commercially produced control material is not available for this assay so it must be produced in-house.

12. wRBC from single donors should be lysed in bulk following the section of protocol ‘prepare hemolysates’.
13. The hemolysate should be divided into 200 μL aliquots and stored in Sarstedt 2 mL microtubes (or similar) at ‒70°C, for analysis with each batch.

**CRITICAL:** Prepare sufficient aliquots of QC for a new aliquot to be run with each batch that is expected to be run in the following year. Run a new preparation of QC material alongside the previous preparation for 10 batches before moving on to using the new preparation.

***Note:*** It is important not to mix donations from different donors because clotting is likely to occur, as in a transfusion reaction.

14. Three levels of QC samples should be prepared.
  a. QC ‘A’: Ideally produced from blood drawn from a riboflavin-deficient donor. If it is not possible to identify a riboflavin-deficient donor, a donor with a high EGRAC should be selected.
  b. QC ‘B’: Should be produced from blood drawn from a donor without riboflavin deficiency.
  c. QC ‘C’: “Low Activity” can be derived from any single-donor human wRBC sample, diluted 1:1 with physiological saline. This is to assess the robustness of the assay.

### Day 0 (Day before analysis)

**Timing: 0.5–2 h**

15. Check enough in-date phosphate buffer and 1% Tween 20 are available and prepare if required – see section ‘materials and equipment’.

### Day 1 (analysis day)

**Timing: 0.5 h**

16. Prepare equipment for use:
  a. Turn on the incubator and allow it to reach 37°C.
  b. Turn on the refrigerated centrifuge and set it to cool to 4°C.
  c. Turn on the plate reader and set the temperature to 28°C.
  d. Take the controls and samples out from ‒70°C storage and mix on a roller mixer until the samples have thawed.

### Prepare hemolysates

**Timing: 1.5 h**

17. After thawing, mix the samples of wRBC gently by inversion.
18. Transfer 1 volume of wRBC to a Sarstedt 2 mL micro tube (or similar) and mix with 2 volumes of deionized water, for example 200 μL wRBC and 400 μL deionized water.
19. Cap and mix thoroughly by inversion, leave on the bench for 10 min at room temperature to allow lysis.

***Note:*** wRBC are sticky and can be difficult to pipette. It is recommended that positive displacement pipettes are used where available. If only standard pipettes are available, then reverse pipetting is recommended. Although only 5 μL of hemolysate is required for the assay it is recommended that volumes of wRBC <50 μL should not be pipetted.

**Pause point:** Lysates may be stored at ‒70°C prior to the assay, if required.

### Plate reader performance check

**Timing: 3 h**

***Note:*** This check can be used to determine the level of within-plate imprecision due solely to the variation of temperature within the incubation chamber of the plate reader. If using the recommended plate reader, this protocol can be followed once a year to ensure optimal performance. This protocol can also be used to assess the suitability of alternative plate readers for this procedure.

20. Remove a vial of QC material with sufficient volume to provide 30 μL hemolysate and mix on a roller mixer until the sample has thawed.
21. Centrifuge the selected hemolysate sample in the refrigerated centrifuge for 10 min at 4000 × *g.*
22. Pipette 30 μL QC material hemolysate and 6.5 mL phosphate buffer into a 50 mL Falcon tube.
23. Add 1.3 mL FAD (to ensure higher rates) and swirl gently to mix.
24. Incubate for 30 min at 37°C.
25. Add 11 mL glutathione solution and swirl gently to mix.
26. Add 3.3 mL NADPH solution and swirl gently to mix.
27. Pipette 200 μL of the mixture into each well of the 96-well plate.
28. Mix on the plate shaker at 600 RPM for 30 s.
29. Inspect wells for bubbles, if present it is advisable to burst it with a clean micropipette tip.
30. Read each well of the plate at 340 nm 40 times at 1 min intervals, with the plate reader set to gently shake the plate for 5 s before each read.
31. The first 20 readings, where the temperature of the plate and its contents equilibrate with that of the reader, are not included in the calculations.
32. Using the subsequent 20 readings, subtract each reading from the previous reading to calculate the change in absorbance at each of the final 20 read points. Calculate the mean of these values to give the mean absorbance change.

***Note:*** An example result calculation is shown in quantification and statistical analysis.

33. Calculate the mean, standard deviation and percent coefficient of variation (%CV) of the change in absorbance measurements for each row, each column and for the whole plate.
34. Inspect the results to look for trends in rate (i.e., in temperature) and also to look for edge effects (where central wells may be at a different temperature to the edge wells).
35. If the %CVs of the rows or columns demonstrate unevenness in the enzyme rate and thus in the temperature in the wells, or if there is an ‘edge effect’, the part of the plate affected is not suitable for this assay. The recommendation is that for rows and columns within the plate the %CV should be <2% and for the plate overall, <3%.
36. Using notional “blank” rates (e.g., average blank rates obtained from QC data), calculate “EGRAC" for each set of four wells as described in the ‘calculation of results’, as would be done for an assay. Ideally each calculated EGRAC will be 1.00 because the same reaction is occurring in every well. Inspect the results for deviations from 1.00; this provides an indication of the within-batch imprecision to be expected from an assay.

## Key resources table

**Table:** 

REAGENT or RESOURCE	SOURCE	IDENTIFIER
**Biological samples**		

EDTA or lithium heparin anticoagulated whole blood (for use as quality control material)	Any supplier	

**Chemicals, peptides, and recombinant proteins**		

Sodium hydroxide	BDH	Cat#BDH9292; CAS: 1310-73-2
Potassium phosphate (KH_2_PO_4_)	Sigma	Cat#P-0662; CAS: 7778-77-0
Ethylenediaminetetraacetic acid (EDTA)	Sigma	Cat#E-1644; CAS: 638-92-6
Oxidized glutathione	Sigma	Cat#G-2140; CAS: 27025-41-8
β-Nicotinamide adenine dinucleotide 2′-phosphate reduced tetrasodium salt hydrate (β-NADPH)	Sigma	Cat#N-6505; CAS: 2646-71-71
Flavin adenine dinucleotide (FAD)	Sigma	Cat#F-6625; CAS: 84366-81-4
Tween 20	Sigma	Cat#P1379; CAS: 9005-64-5
0.9% Saline (NaCl) solution	Any supplier	

**Software and algorithms**		

Microsoft Excel	Microsoft	
SkanIt software for microplate readers	Thermo Fisher Scientific	#5187139

**Other**		

Multiskan FC microplate photometer with incubator	Thermo Fisher Scientific	#51119100
Orbital microplate shaker with digital control	Any supplier	
Safety cabinet	Any supplier	
Incubator with fan	Any supplier	
Refrigerated centrifuge	Any supplier	
Greiner UV-Star 96-well plates	Greiner	#655801
Microplate sealers	Any supplier	
Positive displacement pipette (50–200 μL) with disposable tips	Any supplier	
P20, P200, P1000, and P5000 pipettes	Any supplier	
1 mL Pastettes	Any supplier	
Sarstedt 2 mL microtubes	Sarstedt	
Falcon 5 mL, 15 mL, and 50 mL tubes	Any supplier	
Roller mixer	Any supplier	

## Materials and equipment

**CRITICAL:** This protocol uses a Thermo Fisher Scientific Multiskan FC microplate photometer (with incubator) to measure a decrease in absorbance at 340 nm at a controlled temperature of 28°C. The ability of the photometer to maintain a consistent temperature across the 96-well plate for the duration of the assay must be established using the ‘plate reader performance check’ above.

***Alternatives:*** Other temperature controlled plate readers may be suitable for this assay. We recommend using the ‘plate reader performance check’ described above to assess the suitability of alternative plate readers.

**Table:** 100 mM phosphate buffer

Reagent	Final concentration	Amount
Potassium phosphate (KH_2_PO_4_)	100 mM	13.6 *g*
EDTA		1 *g*
Deionized H_2_O		1 L
10% w/v NaOH		As required
**Total**	**N/A**	**1 L**

• Dissolve 13.6 *g* of potassium phosphate in 800 mL deionized H_2_O.
• Use 10% w/v NaOH to adjust the pH to 7.4.
• Make up to 1 L with deionized H_2_O.

Store at +4°C for up to 1 month.

**CRITICAL:** NaOH is corrosive. Use personal protective equipment (PPE) including eye protection, gloves and lab coat.

**CRITICAL:** EDTA is harmful if inhaled. Use PPE including eye protection, gloves and lab coat and work in a safety cabinet.

**Table:** 10% Tween 20 (intermediate dilution)

Reagent	Final concentration	Amount
Tween 20	10% Tween 20	1 mL
Deionized H_2_O		9 mL
**Total**	**N/A**	**10 mL**

• Measure 9 mL deionized H_2_O into a 15 mL Falcon tube.
• Add 1 mL Tween 20 below the meniscus using a Pastette.
• Use the water meniscus and graduations to determine the volume.
• Cap the tube and mix by inversion.

***Note:*** Do not attempt to pipette neat Tween 20 quantitatively.

Store at +4°C for up to 6 months.

**Table:** 1% Tween 20

Reagent	Final concentration	Amount
10% Tween 20	1% Tween 20	1 mL
Deionized H_2_O		9 mL
**Total**	**N/A**	**10 mL**

• Add 1 mL of 10% Tween 20 into a 15 mL Falcon tube using a pipette.
• Make up to 10 mL with deionized H_2_O.
• Cap the tube and mix by inversion.

Store at +4°C for up to 6 months.

**Table:** 760 μg/mL flavin adenine dinucleotide (FAD) (intermediate dilution)

Reagent	Final concentration	Amount
FAD	760 μg/mL	3.8 *g*
Deionized H_2_O		5 mL
**Total**	**N/A**	**5 mL**

• Weigh 3.8 *g* FAD into a Sarstedt 5 mL screw cap tube.
• Add 5 mL deionized H_2_O.
• Cap the tube and roller mix until dissolved.

Store at +4°C for up to 1 day.

**CRITICAL:** Ensure that this reagent does not contaminate any of the other reagents as this could lead to the contamination of the basal wells.

**Table:** 19 μg/mL flavin adenine dinucleotide (FAD) (assay dilution)

Reagent	Final concentration	Amount
760 μg/mL FAD intermediate dilution	19 μg/mL	50 μL
Deionized H_2_O		4.95 mL
**Total**	**N/A**	**5 mL**

• Pipette 50 μL of the 760 μg/mL FAD intermediate dilution into a Sarstedt screw cap 5 mL tube.
• Add 4.95 mL deionized H_2_O.
• Cap the tube invert 10 times to mix.

Store at +4°C for up to 1 day.

**CRITICAL:** Ensure that this reagent does not contaminate any of the other reagents as this could lead to the contamination of the basal wells.

**Table:** 2 mg/mL glutathione solution

Reagent	Final concentration	Amount
Oxidized glutathione	2 mg/mL	50 mg
1% Tween 20		100 μL
100 mM phosphate buffer		25 mL
**Total**	**N/A**	**25 mL**

• Weigh 50 mg oxidized glutathione into graduated 50 mL Falcon tube.
• Add 100 μL Tween 20.
• Make up to 25 mL with 100 mM phosphate buffer.
• Cap the tube and roller mix until dissolved.

Store at +4°C for up to 1 day.

***Note:*** This dilution provides sufficient glutathione reagent for 2 plates (with some pipetting excess), but can be scaled up as appropriate if more plates are to be run on the day.

**Table:** 0.6 mg/mL NADPH solution

Reagent	Final concentration	Amount
NADPH	0.6 mg/mL	6 mg
100 mM phosphate buffer		10 mL
**Total**	**N/A**	**10 mL**

• Weigh 6 mg oxidized NADPH into graduated 50 mL Falcon tube.
• Make up to 10 mL with 100 mM phosphate buffer.
• Cap the tube and roller mix until dissolved.

Store at +4°C for up to 1 day

***Note:*** This dilution provides sufficient NADPH reagent for 2 plates (with some pipetting excess), but can be scaled up as appropriate if more plates are to be run on the day.

## Step-by-step method details

### Load 96-well plate

**Timing: 1.5–2 h**

In this step the samples and QCs are added to the plate in quadruplicate. For each sample or QC, a pair of wells is spiked with FAD to fully activate EGR. The other pair of wells have only buffer added to maintain sample volume and basal enzyme activity. After incubation, glutathione is added as substrate and NADPH as a reducing agent.

**Figure 1:**
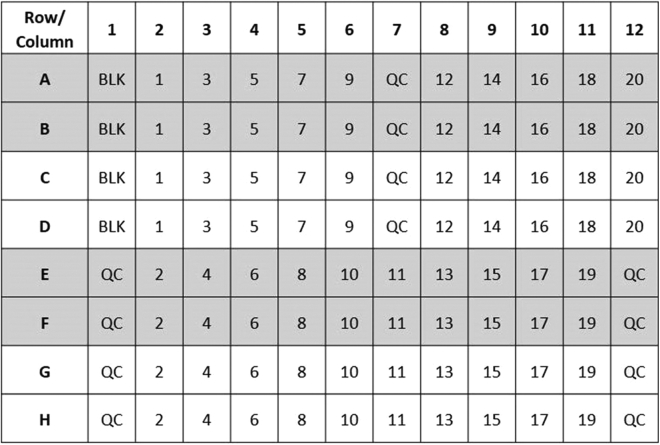
Recommended plate map Abbreviations: BLK, blank; QC, quality control

***Note:*** The samples should be assayed at the positions shown, ensuring that the position of any individual QC is varied from assay to assay. Phosphate buffer is used for the four blanks.

1. Centrifuge the hemolysate samples and controls in the refrigerated centrifuge for 10 min at 4000 × *g.*
2. Produce 200-fold dilutions of the hemolysate samples and controls with phosphate buffer; 5 μL + 1000 μL phosphate buffer in a 2 mL Sarstedt micro tube. Cap and invert 10 times to mix.
3. Pipette 60 μL quadruplicates of each sample, QC and blank as per the plate map in Figure 1.

4. Using a multichannel pipette, add 12 μL phosphate buffer to rows A, B, E, F (basal). Add12 μL FAD-assay dilution to rows C, D, G, H (activated).
5. Cover the plate with a self-adhesive sealer and mix the plate on the plate shaker at 600 RPM for 60 s.
6. Carefully place the plate in the incubator at 37°C for 30 min.
7. Remove plate from incubator. Remove the plate sealer and, using a multichannel pipette, add 100 μL of glutathione solution per well and re-seal with a new plate-sealer.
8. Mix the plate on the plate shaker at 600 RPM for 2 min.
9. Remove the plate sealer. Using a multichannel pipette, add 30 μL of NADPH solution per well and re-seal with a new plate-sealer.
10. Mix the plate on the plate shaker at 600 RPM for 30 s.
11. Remove the plate sealer. Load plate onto the plate reader.

### Read plate

**Timing: 40 min**

In this step the absorbance at 340 nm is measured 40 times in each of the wells at 1 min intervals at a controlled temperature of 28°C. During this time the EGR in the sample catalyzes the reduction of oxidized glutathione to glutathione, utilizing added NADPH as the electron donor. Oxidization of NADPH to NADP causes a measureable change in absorbance at 340 nm that is proportional to the activation of EGR.

$$Glutathione\left(oxidised\right)+NADPH+H^{+}\underset{Glutathione\;reductase}{⇋}Glutathione\left(reduced\right)+NADP$$

12. Read each well of the plate at 340 nm 40 times at 1 min intervals, with reader set to briefly shake the plate for 5 s before each read.

## Expected outcomes

Table 1 shows an example of raw data for an individual sample. The first 20 readings have been omitted as per the protocol.

**Table 1:** Example of raw data measured on a plate reader

Reading	Time (s)	Absorbance measured at 340 nm			
		Basal well 1	Basal well 2	Activated well 1	Activated well 2
21	1200	0.3740	0.3720	0.3680	0.3740
22	1260	0.3700	0.3690	0.3640	0.3700
23	1320	0.3670	0.3650	0.3610	0.3670
24	1380	0.3630	0.3620	0.3570	0.3630
25	1440	0.3590	0.3590	0.3530	0.3590
26	1500	0.3560	0.3550	0.3500	0.3550
27	1560	0.3530	0.3520	0.3460	0.3520
28	1620	0.3490	0.3480	0.3420	0.3480
29	1680	0.3450	0.3450	0.3390	0.3440
30	1740	0.3420	0.3420	0.3350	0.3410
31	1800	0.3390	0.3380	0.3310	0.3370
32	1860	0.3350	0.3350	0.3280	0.3340
33	1920	0.3320	0.3320	0.3240	0.3300
34	1980	0.3290	0.3280	0.3200	0.3260
35	2040	0.3250	0.3250	0.3170	0.3230
36	2100	0.3210	0.3220	0.3130	0.3190
37	2160	0.3180	0.3190	0.3100	0.3160
38	2220	0.3150	0.3150	0.3060	0.3120
39	2280	0.3120	0.3120	0.3030	0.3090
40	2340	0.3080	0.3090	0.3000	0.3060

In our experience from measuring samples from populations with different riboflavin status, mean changes in absorbance per minute can range from ‒0.0003 to ‒0.005 in basal wells and ‒0.001 to ‒0.005 in activated wells. These ranges are specific to the cohorts of samples measured in our laboratory using the methods as described here and can only provide a guide to expected results.

## Quantification and statistical analysis

Following the steps below yields the EGRAC for each of the samples. In the assay, EGR activity with and without the addition of FAD, is measured. The EGRAC reflects the degree of additional stimulation of EGR in the activated wells (i.e., the wells spiked with FAD) compared to that of the basal wells, and therefore because riboflavin is a component of FAD, the assay provides a marker of riboflavin nutritional status. If little additional stimulation of EGR occurs after the addition of FAD this demonstrates relatively high saturation of EGR with endogenous FAD, and an EGRAC close to 1 is obtained, indicating riboflavin sufficiency. Where riboflavin status is inadequate, greater stimulation occurs, and a higher EGRAC is observed.

### Calculation of results

**Timing: 1–1.5 h**

In this step the blank absorbance readings are used to correct the sample and QC absorbance readings. The mean change in absorbance per minute can then be calculated in both the basal and activated wells. After an imprecision check of the duplicate pair readings, the ratio of the basal and activated wells (EGRAC) is calculated.

1. Do not use the first 20 readings, where the temperature of the plate and its contents equilibrate with that of the reader, in the calculations. See example data in Table 1.
2. Calculate the mean change in absorbance per well by subtracting the previous reading from each reading, during the last 20 readings and calculating the average of these as shown in Table 2.

**Table 2:** Example calculation of mean absorbance change using raw data

Reading	Basal well 1	Basal well 2	Activated well 1	Activated well 2
Reading ‘22’ minus ‘21’	-0.0040	-0.0030	-0.0040	-0.0040
Reading ‘23’ minus ‘22’	-0.0030	-0.0040	-0.0030	-0.0030
Reading ‘24’ minus ‘23’	-0.0040	-0.0030	-0.0040	-0.0040
Reading ‘25’ minus ‘24’	-0.0040	-0.0030	-0.0040	-0.0040
Reading ‘26’ minus ‘25’	-0.0030	-0.0040	-0.0030	-0.0040
Reading ‘27’ minus ‘26’	-0.0030	-0.0030	-0.0040	-0.0030
Reading ‘28’ minus ‘27’	-0.0040	-0.0040	-0.0040	-0.0040
Reading ‘29’ minus ‘28’	-0.0040	-0.0030	-0.0030	-0.0040
Reading ‘30’ minus ‘29’	-0.0030	-0.0030	-0.0040	-0.0030
Reading ‘31’ minus ‘30’	-0.0030	-0.0040	-0.0040	-0.0040
Reading ‘32‘ minus ‘31’	-0.0040	-0.0030	-0.0030	-0.0030
Reading ‘33‘ minus ‘32’	-0.0030	-0.0030	-0.0040	-0.0040
Reading ’34‘ minus ‘33’	-0.0030	-0.0040	-0.0040	-0.0040
Reading ‘35‘ minus ‘34’	-0.0040	-0.0030	-0.0030	-0.0030
Reading ‘36‘ minus ‘35’	-0.0040	-0.0030	-0.0040	-0.0040
Reading ‘37‘ minus ‘36’	-0.0030	-0.0030	-0.0030	-0.0030
Reading ‘38‘ minus ‘37’	-0.0030	-0.0040	-0.0040	-0.0040
Reading ‘39’ minus ‘38’	-0.0030	-0.0030	-0.0030	-0.0030
Reading ‘40’ minus ‘39’	-0.0040	-0.0030	-0.0030	-0.0030
**Mean absorbance change**	**-0.0035**	**-0.0033**	**-0.0036**	**-0.0036**
3. Subtract the mean change in absorbance of the blank wells from every basal and activated rate.

***Note:*** In the assay each sample is measured in duplicate with the addition of FAD (activated) and in duplicate without addition of FAD (basal). Duplicate measurements are required to allow interpretation of reproducibility of the pipetting of samples and reagents. Before the EGRAC can be calculated the imprecision of the activated and basal duplicates needs to be assessed; EGRAC should not be calculated if imprecision of duplicate measurements exceeds 10% for either the basal or activated measurement. To calculate imprecision of duplicates use the following formula:

$$\frac{V_{1}-V_{2}}{{((V}_{1}+V_{2})/2))}X100$$

*V*_*1*_ = mean change in absorbance duplicate “1”

*V*_*2*_ = mean change in absorbance duplicate “2”

A duplicate ratio of up to 10% is acceptable. Analysis for samples with a ratio of >10% should be repeated.

4. Calculate the mean rate for each activated and basal duplicate pair and divide the mean rate of the activated duplicate wells by the mean rate of the basal duplicate wells to calculate the EGRAC as shown in Table 3.

**Table 3:** Example calculation of EGRAC

	Mean absorbance change at 340 nm
Basal well 1	-0.0035
Basal well 2	-0.0033
**Mean basal**	**-0.0034**
Activated well 1	-0.0036
Activated well 2	-0.0036
**Mean activated**	**-0.0036**
**Mean activated/mean basal (EGRAC)**	**1.05**
5. For QC samples, keep a record of values for each batch. Calculate the mean, standard deviation and %CV for each QC sample. After the first 10 batches, a +/- 2 standard deviation range can be calculated and applied using Westgard^2^ or local rules to assess the validity of a batch.

## Limitations

The use of the EGRAC ratio for assessment of riboflavin status assumes that endogenous riboflavin will be the limiting factor on the reaction in the basal wells. Although rare, it is possible that in some individuals the availability of EGR may be reduced.^3^ In these individuals a suboptimal level of endogenous riboflavin would saturate the available EGR and in the EGRAC assay a ratio close to “1” would be measured. A similar scenario could be encountered if the EGR had degraded prior to analysis. EGRAC is not a suitable test of riboflavin status in people with glucose-6-phosphate dehydrogenase (G6PD) deficiency because GR retains FAD leading to higher GR activity and low EGRAC.^4^ Prevalence of G6PD deficiency varies globally, with the highest rates up to 7.5% observed in the Middle-East and areas in sub-Saharan Africa.^5^ β-thalassemia, protein-energy malnutrition and severe hypothyroidism may also influence interpretation of EGRAC.^5^

Cut-offs of EGRAC that indicate riboflavin deficiency are poorly defined and have ranged between >1.2 to >1.7.^5^ The European Food Safety Authority (EFSA) concluded that EGRAC of <1.3 indicates adequate riboflavin status.^4^

Methodological differences in assay conditions (such as FAD concentration and incubation times) can influence EGRAC values,^6^ underlining the need for availability of detailed method information and standardization of procedures. Few studies have reported on EGR stability after specimen collection, either in whole blood or hemolysates. EGRAC measured in whole blood kept at 4°C appears to be relatively stable up to 4 h, and as long as 24 h, after collection.^7^^,^^8^ In contrast, lysed cells displayed decreasing enzyme stability as storage temperature was increased.^7^ Comparisons in our laboratory suggest either EDTA or lithium heparin whole blood may be used to measure EGRAC.^8^

## Troubleshooting

### Problem 1: Duplicate imprecision >10%

Most problems with the assay will be revealed when calculating imprecision of the basal or activated duplicates during step 3 of quantification and statistical analysis. If there is a difference of >10% in any of the duplicates of QC samples or test samples the EGRAC should not be calculated for the affected sample. Imprecision can be caused at almost every step of the assay so it is important to double check that the protocol is being followed carefully. Good pipetting technique is important and this should be the first consideration when troubleshooting.

If > 3 samples on a plate show a difference of >10% this would indicate a fundamental problem with either the preparation of the plate or the plate reader, in this instance it is recommended that the analysis of the whole plate is repeated.

### Potential solution

**Figure 2:**
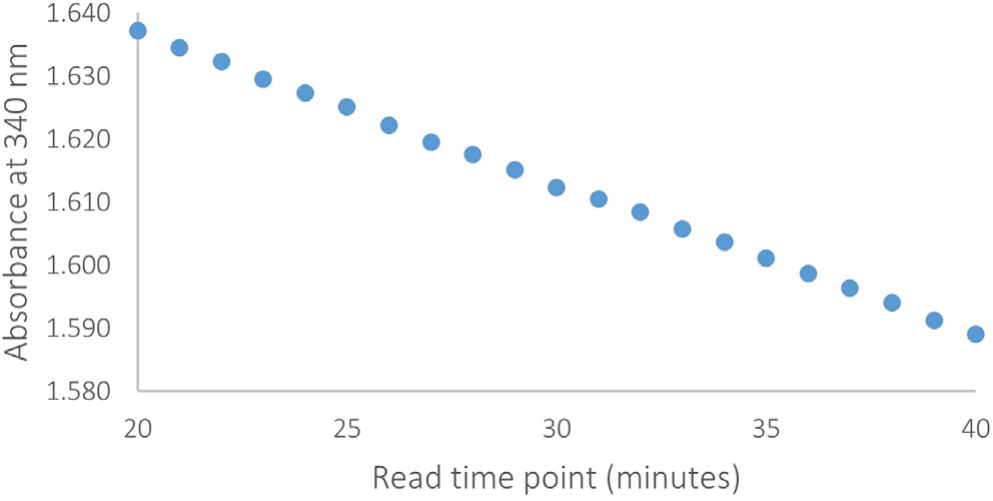
Absorbance over time in a sample with normal reaction kinetics

**Figure 3:**
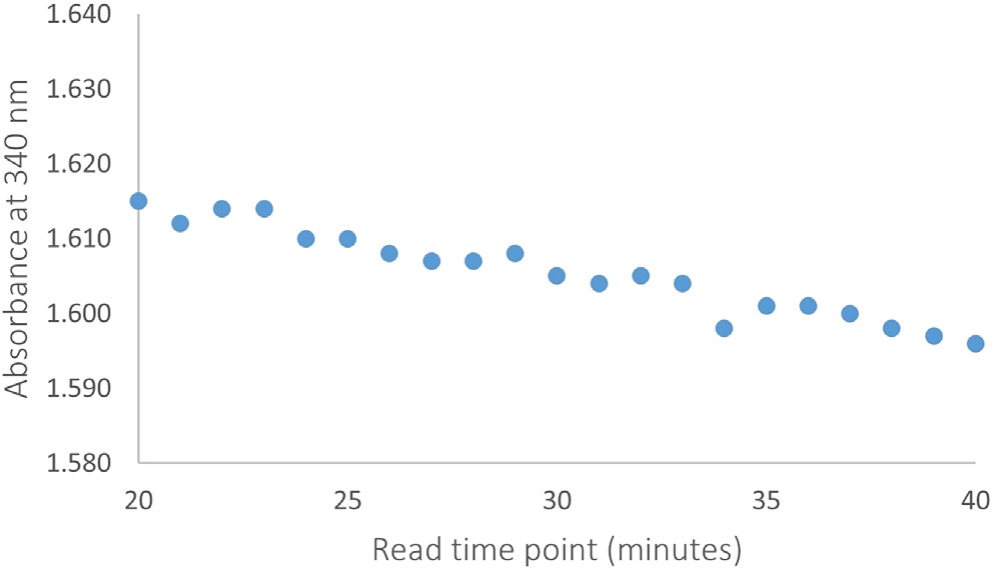
Absorbance over time in a sample with abnormal reaction kinetics

When troubleshooting it can also be useful to plot the change in absorbance over time in the final 20 read points for each of the well positions on a plate. For example, Figure 2 shows the absorbance over time in a sample with normal reaction kinetics. Figure 2 shows a relatively consistent decrease in absorbance over time and is an example of how the assay should perform under normal circumstances. Figure 3 shows an inconsistent decrease in absorbance over time that could be caused by

• Particulates in the reaction mixture.
• Electrical interference (although this would usually be seen as spikes in the kinetic trace).
• Poor plate reader performance (consider running a plate reader performance check, as above).

### Problem 2: QC results outside the defined range

If when following step 5 of quantification and statistical analysis the measured ratio in a single QC level falls outside the +/- 2 standard deviation (SD) limits calculated, this indicates that the results associated with the batch should be interpreted with caution and the cause investigated. The causes could be due to poor pipetting or abnormal reaction kinetics (see above). If more than one QC falls outside the +/-2 SD limits the results should be discarded and the cause investigated before repeating the batch.

### Potential solution

• May indicate the need to repeat the batch, according to local procedures.
• Review/check pipette performance and pipetting technique.

## Resource availability

### Lead contact

Further information and requests for resources and reagents should be directed to and will be fulfilled by the lead contact, Kerry Jones (kerry.jones@mrc-epid.cam.ac.uk).

### Materials availability

We recommend the Thermo Fisher Scientific Multiskan FC microplate photometer with incubator (part number: 51119100) for this assay. It may be possible to substitute this for an alternative plate reader with both an incubator and the capability to read a 96-well plate at 340 nm. We recommend following the “Plate reader performance check” as described above.

All chemical reagents can be substituted for their equivalent from alternative suppliers.

We would be willing to share and exchange QC materials for purposes of validation and interlaboratory comparison.

### Data and code availability

There are no data or code associated with this protocol.
